# Neurofeedback for COVID-19 Brain Fog: A Secondary Analysis

**DOI:** 10.7759/cureus.79222

**Published:** 2025-02-18

**Authors:** JeAnnah Rose AbouAssaly, Tatsuya Masuko, Harue Sasai-Masuko, Frederick Strale

**Affiliations:** 1 Neurofeedback, The Oxford Center, Brighton, USA; 2 Orthopedic Surgery, Emerald Orthopedic & Pain Clinic, Sapporo, JPN; 3 Internal Medicine, Emerald Orthopedic ＆ Pain Clinic, Sapporo, JPN; 4 Biostatistics, The Oxford Center, Brighton, USA

**Keywords:** brain fog, covid-19, mild-to-severe cognitive dysfunction, neurofeedback therapy, quantitative eeg (qeeg), z-score neurofeedback

## Abstract

This secondary analysis technical report uses quantitative electroencephalography (qEEG) neurofeedback to treat brain fog, a group of cognitive challenges such as sluggish thinking, memory lapses, confusion, and poor focus, often observed in long COVID. In the original case report, a 34-year-old female patient was administered approximately 15 sessions of Z-score neurofeedback. Pretest and post-test neurofeedback training qEEG reported significant declines in high-beta activity, i.e., beta, Hi beta, beta 1, and beta 3, and notable increases in alpha and theta brainwaves. These improvements were most evident at electrode sites C4, F3, and P3. The patient reported improved attention and memory, reduced anxiety, and less pain, although depression reduction was less. These results suggest that a relatively short program of neurofeedback can assist in shifting the brain from hyperarousal to a calmer, more efficient state. This may potentially offer a non-pharmacological method for treating constant post-COVID brain fog. Additional studies are necessary to validate these findings.

## Introduction

Although there is currently no formal medical consensus definition of the condition widely referred to as “COVID-19 brain fog,” the symptoms identified in the existing research can simply be outlined. These manifestations commonly include a slower or more clouded thought process, problems remembering information, disorientation episodes, and persistent difficulty concentrating [1]. Other reported issues may involve trouble finding the right words, reduced mental stamina, and a general sense that cognitive tasks require more effort than usual. Following the COVID-19 outbreak, an increasing number of patients have experienced COVID-19 brain fog. Since these symptoms indicate brain dysfunction, an electroencephalogram (EEG) helps evaluate brain conditions due to its convenience [1]. 

The term “brain fog” is becoming more prevalent, mainly as we uncover the long-term effects of COVID-19. While common in other autoimmune disorders, brain fog remains poorly understood. Currently, there is no effective treatment, leaving many individuals to cope with often debilitating symptoms. Recent studies suggest that quantitative electroencephalography (qEEG) can reveal distinct brain activity patterns in individuals with post-COVID brain fog compared to those unaffected by COVID-19 [2]. 

A recent systematic review by Gorenshtein et al. examined various interventions to alleviate long-term COVID-19 brain fog, which is characterized by confusion, short-term memory loss, and difficulty concentrating. Seventeen studies met inclusion criteria, encompassing four broad treatment approaches [3]. 

With rehabilitation strategies, various multifaceted rehabilitation programs were employed. Results differed, but many approaches demonstrated improvements in cognitive function. With noninvasive brain stimulation, multiple investigations have explored methods such as transcranial magnetic stimulation [3]. All reported enhancements in participants’ cognitive abilities. With hyperbaric oxygen therapy (HBOT), several evaluations were performed on HBOT. Positive changes were observed in cognitive assessment scores and brain perfusion. With Palmitoylethanolamide and Luteolin (PEA-LUT), some findings indicated that PEA-LUT supplementation contributed to cognitive improvements [3]. 

Overall, noninvasive brain stimulation and HBOT showed promise for restoring cortical excitability and improving perfusion in patients experiencing long-term brain fog. Rehabilitation strategies and PEA-LUT supplementation also demonstrated beneficial effects. Future research should examine multimodal interventions and incorporate more extended follow-up periods better to understand these improvements' sustainability [3]. 

Bilali et al. reviewed 10 studies, and the findings highlight the need for a holistic approach to treating post-COVID brain fog. Healthcare workers must thoroughly investigate the biological and psychosocial factors contributing to brain fog symptoms to deliver appropriate care. However, further scientific research is essential to evaluate the effectiveness of treatment methods for public health and well-being [4]. 

Since the initial SARS-CoV-2 case emerged in late 2019 and the first scientific reports of brain fog appeared in October 2020, only three randomized controlled trials (RCTs) on brain fog have met the required criteria. Additional limitations included the small sample sizes of the studies reviewed, possible lack of data quality and blinding, and the limited number of alternative treatments available, such as aromatherapy and psychoeducation [4].

Effects of neurofeedback on brain fog 

Masuko and Masuko commented that neurofeedback has proven effective for various common disorders and symptoms, such as anxiety, depression, headaches, and pain. Additionally, it is reported to enhance cognitive functions, including processing speed and executive functions like attention, planning, organization, problem-solving, and overall performance [5]. 

Luckos et al. reported performance before and after approximately 15 weeks of neurofeedback training (theta/beta and sensory-motor rhythm (SMR) protocols on C3/C4) with a 48-year-old female patient who contracted COVID-19 in October 2020 and developed a range of severe symptoms including intense headaches, body aches, loss of smell (anosmia), loss of taste (ageusia), hearing issues, shortness of breath, and chest pain. Although her brain MRI showed no acute lesions, she experienced persistent “brain fog” (neurocognitive impairments) for months afterward, to the point that she was unable to live independently [6]. 

She underwent neurotherapy, specifically EEG neurofeedback combined with goal-oriented cognitive training, to address her long-term cognitive dysfunctions (often called “NeuroCOVID-19”). Her symptoms improved substantially after treatment, allowing her to return to work. The findings suggest that this form of neurotherapy may be a promising approach for alleviating persistent cognitive and neurological symptoms in patients recovering from COVID-19 [6]. 

Substantial gains in IQ were reported, with the patient’s full‐scale, verbal, and nonverbal IQ scores rising from well below average to borderline or near‐average levels [6].

Memory and attention improvements pointed toward notable spatial span boost (from 1st to 75th percentile), immediate and delayed recall, and visuospatial skills. Executive function gains resulted in faster and more accurate trail making and stroop performance, fewer perseverative errors on WCST, and a better ability to maintain and shift sets. With language and comprehension, a dramatic drop in Boston Naming Test errors and improved Token Test scores reflect enhanced naming and auditory comprehension skills [6]. 

Taken together, these changes strongly suggest that the theta/beta and SMR neurofeedback protocols improved multiple aspects of cognitive functioning, especially attention, working memory, executive control, language, and overall intellectual performance. While some scores remain below average, the patient’s shift from the severely impaired range in almost every domain is clinically meaningful and points to significant therapeutic benefit [6]. 

The lack of published studies on qEEG-neurofeedback for COVID-19 brain fog or general brain fog underlines an essential gap in the current scientific literature. Given the broad impact of cognitive impairment on daily functioning and quality of life and the potential of neurofeedback to modulate brain networks in a targeted, non-pharmacological manner, well-designed research is urgently needed. Studies that focus on these areas will help establish whether qEEG-guided neurofeedback is a viable, evidence-based intervention for individuals struggling with long-term or recurrent brain fog symptoms. 

## Technical report

Original study (Masuko & Masuko, 2024) overview

Masuko and Masuko presented a case study of a 34-year-old woman who achieved significant relief from brain fog through neurofeedback. Three months after the COVID-19 infection, the patient started an original treatment method for brain fog using neurofeedback for a total of 15 sessions [5]. 

The patient had no risk factors such as obesity, diabetes, or hypertension. After a fever lasting only one day, she developed various symptoms, such as general fatigue, cough, breathlessness, smell disorder, and taste disorder. Although the treatment team did not understand why another physician prescribed escitalopram oxalate, a selective serotonin reuptake inhibitor, it could not improve her disabilities and she came to the Masuko & Masuko clinic [5].

The patient was administered three traditional Japanese and Chinese Kampo medicines using natural ingredients like plants, minerals, and animal products. These medicines are used to treat various health conditions and are regulated by the Japanese government for safety and efficacy. After taking the Kampo medicines, TJ9 shosaikoto, TJ-41 hochuekkito, and TY-028 keishikakobokukyoninto, prescribed by the clinic for two weeks, most of the patient's symptoms gradually improved [5].

However, significant general fatigue, headache, pins and needles feelings, and brain fog remained, and she could not return to work. Although all results of blood examination, neurological examination, and MRI of the brain and cervical spinal cord were evaluated as normal, the patient’s condition had still not improved a month after COVID-19 infection. At three months post-COVID-19 infection, the patient started an original treatment method for brain fog using neurofeedback for a total of 15 sessions [5].

Original study methods

Neurofeedback Equipment

The Z-score neurofeedback system employed a ProComp Infiniti™ Encoder (Thought Technology Ltd., Montreal, Canada) as its neurofeedback recorder. The procedure was managed using an integrated software package called “Z-Score 6 Suite” (also from Thought Technology Ltd.), which includes both a measurement module (“Report - Z-score indices”) and a treatment module (“Z-score index training”). The EEG data were captured with an EEG-Z™ sensor. In addition, the setup incorporated a TT-EEG 4 Channel Connectivity Kit, NuPrep EEG Skin Prepping Gel, and Ten-20 Conductive EEG Paste-all provided by Thought Technology Ltd. in Montreal [5].

Overview of Z-Score Neurofeedback

Z-score neurofeedback is a technique that compares a patient’s real-time EEG data with a normative database maintained by Applied Neuroscience Inc. (St. Petersburg, USA). An algorithm within the Z-score software computes the deviation (Z-score) from the norm based on the NeuroGuide™ database. The analysis includes six primary metrics: absolute power, relative power, amplitude asymmetry, coherence, phase, and ten power ratios (delta/theta, delta/alpha, delta/beta, delta/Hi beta, theta/alpha, theta/beta, theta/Hi beta, alpha/beta, alpha/Hi beta, and beta/Hi beta) [5].

Brainwave activity is divided into eight frequency bands - Delta: 1-4 Hz, Theta: 4-8 Hz, Alpha: 8-12 Hz, Beta: 12-25 Hz, Hi Beta: 25-30 Hz, Beta1: 12-15 Hz, Beta2: 15-18 Hz, and Beta3: 18-25 Hz [5].

As a result, the four-channel Z-score neurofeedback treatment yields 248 distinct output values. Sensor placements on the scalp, with the earlobe used as the ground, follow the International 10-20 System-where odd numbers designate positions in the left hemisphere and even numbers indicate positions in the right hemisphere [5].

Z-Score Neurofeedback Protocol

A brief explanation of the Z-score neurofeedback treatment was provided, and informed consent was secured prior to starting the therapy. The treatment was delivered in two rounds. With round one, sensors were positioned at the scalp locations C3, C4, T3, and T4. In round two, the sensors were attached at F3, F4, P3, and P4 [5].

Because the four-channel Z-neurofeedback procedure was performed twice in succession, the method is sometimes referred to as the “two-by-four 8-channel” approach. With the first round, sensors were affixed to the patient’s scalp at C3, C4, T3, and T4. The patient’s EEG was recorded with eyes closed using the “Report - Z-score indices” protocol. With the patient’s eyes open, a 10-minute session of “Z-score index training” was conducted at the same sites. During this session, the patient simply observed the screen [5].

Initially, the thresholds were set at ±2.0 standard deviations (SD). Beginning with the 13th session, as the patient’s symptoms improved, the thresholds were adjusted to ±1.0 SD. When the raw Z-score fell within the set threshold, the displayed number turned green. If the raw Z-score exceeded the threshold, it appeared in red or yellow. A reward was provided in the form of moving images on the screen when the average of all raw Z-scores exceeded the preset Z-score index. If the average did not meet the threshold, the images ceased moving, and no reward was given. After training, another eyes-closed EEG was recorded at C3, C4, T3, and T4 [5].

With the second round, the same procedures described above were replicated with sensors positioned at F3, F4, P3, and P4. The entire neurofeedback session lasted approximately 45 minutes, with a total of 15 sessions scheduled at a frequency of one or two sessions per week [5].

EEG Measurement and Analysis

Total values for each EEG channel were recorded before the first session and again before the 15th session using the “Report - Z-score indices” tool. The percentage decrease was calculated using the following formula: 

$\%\text{ Decrease} = \left(\frac{\text{Total value before 1st session} - \text{Total value before 15th session}}{\text{Total value before 1st session}}\right) \times 100$ [5].

Asymmetry refers to the imbalance between two corresponding sites (e.g., C3 vs. C4) as defined by the International 10-20 System. This measurement is particularly valuable for treatment evaluation because, for example, a higher alpha value in the left hemisphere compared to the right is associated with depression, whereas increased beta values in the right hemisphere or widespread high beta activity suggest anxiety [5].

Step 1: The difference in total values between homologous sites (C3-C4, T3-T4, F3-F4, and P3-P4) was calculated for both the pre-treatment and post-treatment sessions [5].

Step 2: For each wavelength at each site, values were recorded before the first session and before the 15th session. The percentage increase was computed using the formula: 

$\%\text{ Increase at a Site} = \left(\frac{\text{Value before 15th session} - \text{Value before 1st session}}{\text{Value before 1st session}}\right) \times 100$ [5].

Finally, for each wavelength, the percentage increase across all sites was determined by the following calculation: 

$\%\text{ Increase (wavelength)} = \left(\frac{\text{Total value after session} - \text{Total value before session}}{\text{Total value before session}}\right) \times 100$ [5].

Original study outcomes

Pain and psychological evaluations indicated that her discomfort diminished, and her anxiety subsided. Concurrent qEEG findings highlighted several notable patterns: C4 appeared to be the region most severely impacted by brain fog but showed the greatest post-treatment improvement [5]. In addition, alpha-wave activity increased at nearly all sites, while beta 1, beta 2, beta 3, and high-beta activity decreased. Remarkably, theta and alpha frequencies rose after the first and second sessions, whereas higher-beta frequencies (beta 3 and high-beta) consistently declined throughout every session [5]. 

Although the patient’s anxiety scores improved to a greater extent than her depression scores, these results corroborated the initial assessments, indicating that her baseline levels of depression were higher than her anxiety. Again, C4 emerged as the critical site: it demonstrated the lowest percentage reduction overall and recorded the slightest difference in total values at C3-C4 before the 15th session [5]. The general trend of increased alpha waves and decreased beta waves observed after neurofeedback treatment aligned closely with the patient’s psychological measurements. Notably, whereas neurofeedback protocols for conditions like attention deficit hyperactivity disorder, anxiety, depression, and insomnia often demand 30-40 sessions to achieve comparable outcomes, this patient exhibited marked increases in theta and alpha activity, as well as decreases in higher-beta ranges, beginning as early as the first and second sessions [5]. 

In Table 1, the total values at F3, F4, P3, P4, T3, and T4 all diminished, while C3 and C4 increased. C4 exhibited the smallest percentage decrease, suggesting it was both the site most affected by brain fog and the one displaying the most pronounced recovery. Brain fog widened the differences among homologous site pairs, but these discrepancies diminished following neurofeedback. The smallest subtraction value at C3-C4 further supports the idea that these regions are most amenable to neurofeedback interventions, an observation aligning with prior reports that link C3 and C4 to depression and anxiety [5]. 

**Table 1:** Each value (microvolt) at each wavelength at all sites and the total values at all wavelengths at each site before the first session and before the 15th session (Masuko & Masuko, 2024). This table is attributed to, and permission was obtained from the authors of the originally published article [5]. This is not required only in cases where the table is published under a Creative Commons License.

Brainwave	C3Pre	C4Pre	T3Pre	T4Pre	F3Pre	F4Pre	P3Pre	P4Pre	C3Post	C4Post	T3Post	T4Post	F3Post	F4Post	P3Post	P4Post
DeltaµV	7.39	6.14	7.08	0	8.7	7.65	7.63	0	6.88	6.54	6.32	0	7.1	5.68	6.03	0
ThetaµV	9.18	7.75	7.83	6.74	9.95	8.73	8.97	8.1	10.6	10.43	9.22	8.34	10.48	8.42	8.56	10.46
AlphaµV	10.77	9.13	9.27	8.21	12.38	10.79	12.02	12.32	13.25	13.23	11.15	10.8	12.52	11.55	11.47	12.37
BetaµV	8.39	6.95	8.62	7.12	8.47	7.43	8.55	8.11	7.79	7.44	7.34	6.49	7.48	7.03	7.78	7.15
HiBetaµV	3.43	2.66	4.33	3.08	3.13	2.53	2.91	2.66	2.68	2.49	2.63	2.17	2.4	2.1	2.41	2.23
Beta1µV	4.97	4.2	4.64	4.06	5.04	4.48	5.06	4.77	4.53	4.38	4.14	3.85	4.48	4.09	4.69	4.36
Beta2µV	4.48	3.66	4.4	3.64	4.39	3.9	4.6	4.4	4.42	4.17	4.09	3.66	4.19	4.02	4.54	3.95
Beta3µV	5.17	4.21	5.74	4.43	5.07	4.35	5.17	4.83	4.78	4.37	4.59	3.84	4.42	4.03	4.46	4.25
Total	53.78	44.7	51.91	37.28	57.13	49.86	54.91	45.19	54.93	53.05	49.48	39.15	53.07	46.92	49.94	44.77
% decrease									-2.14	-18.68	4.68	5.02	7.11	5.9	9.05	0.93

Additionally, per Table 2, the proportion of theta and alpha activity rose substantially at most sites, whereas delta, beta 1, beta 2, beta 3, and high-beta frequencies declined. Higher beta frequencies (beta 3 and high-beta) displayed especially marked reductions, a finding consistent with a relaxed mental state (as reflected by increased alpha) and a decrease in stress-related arousal (as evidenced by lower higher-beta values). These EEG improvements paralleled reductions in self-reported pain, anxiety (PASS-20 and HADS-A), and depression (SDS and HADS-D) [5]. 

**Table 2:** Each value (microvolt) at each wavelength at all sites before the first session and before the 15th session (Masuko & Masuko, 2024). This table is attributed to, and permission was obtained from the authors of the originally published article [5]. This is not required only in cases where the table is published under a Creative Commons License.

Electrode Site	DeltaPre	ThetaPre	AlphaPre	BetaPre	HiBetaPre	Beta1Pre	Beta2Pre	Beta3Pre	DeltaPost	ThetaPost	AlphaPost	BetaPost	HiBetaPost	Beta1Post	Beta2Post	Beta3Post
C3	7.39	9.18	10.77	8.39	3.43	4.97	4.48	5.17	6.88	10.6	13.25	7.79	2.68	4.53	4.42	4.78
C4	6.14	7.75	9.13	6.95	2.66	4.2	3.66	4.21	6.54	10.43	13.23	7.44	2.49	4.38	4.17	4.37
T3	7.08	7.83	9.27	8.62	4.33	4.64	4.4	5.74	6.32	9.22	11.15	7.34	2.63	4.14	4.09	4.59
T4	0	6.74	8.21	7.12	3.08	4.06	3.64	4.43	0	8.34	10.8	6.49	2.17	3.85	3.66	3.84
F3	8.7	9.95	12.38	8.47	3.13	5.04	4.39	5.07	7.1	10.48	12.52	7.48	2.4	4.48	4.19	4.42
F4	7.65	8.73	10.79	7.43	2.53	4.48	3.9	4.35	5.68	8.42	11.55	7.03	2.1	4.09	4.02	4.03
P3	7.63	8.97	12.02	8.55	2.91	5.06	4.6	5.17	6.03	8.56	11.47	7.78	2.41	4.69	4.54	4.46
P4	0	8.1	12.32	8.11	2.66	4.77	4.4	4.83	0	10.46	12.37	7.15	2.23	4.36	3.95	4.25

Nevertheless, while pain and anxiety showed clear improvement, depression scores exhibited fewer substantial gains. The improvements in pain were significant, with SF-MPQ-2 and PDAS scores increasing by 100% and 50%, respectively, following Z-score neurofeedback treatment. Similarly, anxiety levels showed notable enhancement, as evidenced by 100% and 50% improvements in PASS-20 and HADS-A scores. In contrast, depression exhibited only modest gains, with SDS and HADS-D improving by 12.7% and 12.5%, respectively, indicating that the treatment had a limited effect on depressive symptoms [5].

Differences in alpha activity between the left and right hemispheres explain this pattern. Before treatment, alpha values at C3 exceeded those at C4, a finding generally linked to depressive symptoms [5]. After Z-score neurofeedback, the disparity between C3 and C4 nearly vanished, dropping from 1.64 to 0.02, yet the patient’s overall depressive state did not fully resolve, likely because it was intrinsically tied to the underlying brain fog. Meanwhile, anxiety remained minimal, given that the higher-beta values at C4 were not elevated relative to C3 (Table 1). Hence, while neurofeedback effectively modulated EEG patterns associated with brain fog, its ultimate impact on depression was constrained by the patient’s baseline condition [5]. 

The rationale for secondary analysis 

Masuko and Masuko report apparent changes (differences) in EEG measurements across multiple cortical sites but do not report results of any statistical hypothesis tests to determine statistically significant differences between pretest (before the first session) and post-test (before the 15th session) [5].

Although these qualitative findings are compelling, establishing the statistical significance of changes from pre- to post-neurofeedback (particularly following 15 sessions) is crucial for a genuinely evidence-based conclusion. Employing a paired t-test or a non-parametric alternative such as the Wilcoxon signed-rank test would allow for a rigorous comparison of the patient’s pre- and post-intervention measures. By assessing whether observed differences are clinically noteworthy and statistically meaningful, researchers and clinicians can provide more substantial, confirmatory support for the effectiveness of Z-score neurofeedback in alleviating brain fog-related symptoms. 

General objectives 

This study will extend the work using the dataset provided in Masuko and Masuko [5] by inferentially comparing (C3Pre-C3Post), (C4Pre-C4Post), (T3Pre-T3Post), (T4Pre-T4Post), (F3Pre-F3Post), (F4Pre-F4Post), (P3Pre-P3Post), and (P4Pre-P4Post) to ascertain statistically significant differences between Pretest (Before 1st session) and Posttest (Before 15th session). 

This study will also examine for statistically significant differences between (DeltaPre-DeltaPost), (ThetaPre-ThetaPost), (AlphaPre-AlphaPost), (BetaPre-PetaPost), (HiBetaPre-HiBetaPost), (Beta1Pre-Beta1Post), (Beta2Pre-Beta2Post), and (Beta3Pre-Beta3Post). 

Statistical analysis 

Quantitative data analysis was carried out utilizing the Wilcoxen Rank Sum Test, as it was determined that the data was non-normal (skewed). This analysis used IBM SPSS Statistics for Windows, Version 29 (Released 2023; IBM Corp., Armonk, New York, United States). [7]. Alpha (α) was set at 0.05. The null hypothesis was rejected if p-values were less than 0.05, implying a statistically significant difference. 

Results 

Table 3 presents descriptive and inferential statistics for electrode-specific scalp sites.

**Table 3:** Electrode site-specific descriptive and inferential results *Wilcoxon Signed-Rank Test SD: Standard Deviation

Electrode Site	Mean	SD	Median	Skew	Mean Difference	Median Difference	p-value*
C3Pre	6.7225	2.59115	6.28	0.328			
C3Post	6.8663	3.56302	5.83	0.856	-0.14375	0.45	0.674
C4Pre	5.8575	2.25184	5.175	0.329			
C4Post	6.6313	3.62581	5.46	0.934	-1.04375	-0.285	0.025
T3Pre	6.4887	1.97945	6.41	0.194			
T3Post	6.185	2.90241	5.455	0.663	0.30375	0.955	0.575
T4Pre	4.66	2.63617	4.245	-0.393			
T4Post	4.8938	3.4723	3.845	0.498	-0.23375	0.4	0.997
F3Pre	7.1413	3.20477	6.77	0.397			
F3Post	6.6337	4.4602	5.79	0.691	0.5075	0.98	0.05
F4Pre	6.2325	2.83387	5.955	0.329			
F4Post	5.865	3.03164	4.885	0.893	0.3675	1.07	0.161
P3Pre	6.8638	2.96004	6.4	0.496			
P3Post	6.2425	2.88545	5.36	0.701	0.62125	1.04	0.012
P4Pre	5.6488	3.78748	4.8	0.408			
P4Post	5.5962	4.15167	4.305	0.552	0.0525	0.495	0.31

Table 4 represents descriptive and inferential statistics for specific brain wave variables.

**Table 4:** Brainwave-specific descriptive and inferential results *Wilcoxon Signed-Rank Test SD: Standard Deviation

Brainwave	Mean	SD	Median	Skew	Mean Difference	Median Difference	p-value
DeltaPre	5.5738	3.5119	7.235	0.752			
DeltaPost	4.8188	3.00758	6.175	0.752	0.755	1.06	0.046
ThetaPre	8.4063	1.00267	8.415	0.752			
ThetaPost	9.5638	1.02806	9.825	0.752	-1.1575	-1.41	0.036
AlphaPre	10.6112	1.59683	10.78	0.752			
AlphaPost	12.0425	0.93459	11.96	0.752	-1.43125	-1.18	0.036
BetaPre	7.995	0.68218	8.25	0.752			
BetaPost	7.3125	0.42667	7.39	0.752	0.6425	0.86	0.025
HiBetaPre	3.0913	0.58195	2.995	0.752			
HiBetaPost	2.3888	0.21054	2.405	0.752	0.7025	0.59	0.012
Beta1Pre	4.6552	0.38108	4.705	0.752			
Beta1Post	4.315	0.27198	4.37	0.752	0.3375	0.335	0.017
Beta2Pre	4.1838	0.38685	4.395	0.752			
Beta2Post	4.13	0.2737	4.13	0.752	0.05375	0.265	0.483
Beta3Pre	4.8713	0.51798	4.95	0.752			
Beta3Post	4.3425	0.30046	4.395	0.752	0.52875	0.555	0.017

## Discussion

The present secondary analysis extended the original findings of Masuko and Masuko [5] by focusing on two key aspects of the Z-score qEEG neurofeedback intervention for post-COVID-19 “brain fog”: (a) electrode site-specific outcomes (Table 3) and (b) brainwave-frequency-specific outcomes (Table 4). The results may provide supportive evidence that Z-score neurofeedback can address core brain fog symptoms, attenuate maladaptive beta activity, and enhance alpha and theta activity, likely contributing to improved cognitive and affective regulation [8-10].

Electrode site-specific effects

Table 3 presents a detailed summary of Wilcoxon Signed-Rank Test outcomes for eight electrode sites (C3, C4, T3, T4, F3, F4, P3, P4). Each site was evaluated regarding pre- and post-treatment means, standard deviations, medians, skew values, and the p-values associated with the rank tests. The following section highlights the statistically significant findings, interprets their clinical relevance, and discusses the revised total for T4.

Significant changes at C4, F3, and P3

C4 (p = 0.025)

Notably, C4 was one of the sites showing a significant difference from pre- to post-treatment, a finding consistent with Masuko and Masuko’s [5] earlier conclusion that C4 might be a “hot spot” for post-COVID brain fog. The mean difference reported in Table 3 (i.e., −1.04375) suggests that the pre- to post-change was robust enough to reach significance. This aligns with Masuko & Masuko's original case report [5], in which C4 was posited to have the largest percentage decrease in qEEG parameters after neurofeedback. Functionally, the C4 region overlaps with the right sensorimotor cortex and insula [10]. Improvements here may help mediate both cognitive and emotional components of the brain, particularly anxiety and attentional problems in which the sensorimotor strip (SMR) is intimately involved in motor planning, bodily self-awareness, and integration of sensory input [10,11].

F3 (p = 0.05)

The frontal region (F3: left frontal cortex) approached conventional statistical significance in this study, suggesting that part of the intervention’s positive effect might be the restoration of normal frontal-lobe functioning. The frontal lobes, mainly F3 and F4, have long been implicated in executive functions, working memory, and mood regulation [8,9]. As brain fog involves deficits in attention and short-term memory [12], incremental improvements at F3 likely assisted with better cognitive processing, increased attentional capacity, and enhanced mood stabilization.

P3 (p = 0.012)

P3 (left parietal region) showed significant change as well. As the parietal lobes are critically involved in integrating sensory information, visuospatial processing, and aspects of attention [8], the improvements at P3 are consistent with the broader theme that Z-score neurofeedback may help recalibrate higher-level integrative functions. Participants often report fewer “mental cloudiness” complaints when the parietal lobes regain typical activity patterns [5].

Nonsignificant or less pronounced changes at other sites

C3, T3, T4, F4, and P4

Although these sites did not reach conventional significance, the direction of changes was generally aligned with clinical improvements. For instance, at T4, the mean difference was −0.23375 (p = 0.997), suggesting minimal change. However, it is vital to note the discrepancy in the T4 total from the original Table 1 in Masuko and Masuko [5]. The originally published T4 total pretest value of 51.91 was corrected to 37.28 in this secondary analysis, reducing the reported percentage decrease from 24.58% to 5.02%. This correction implies that T4 changes were not as large as originally interpreted, but they remain consistent with a small but positive recalibration of temporal lobe activity. The temporal lobes, particularly T3 and T4, are essential for memory encoding/retrieval and emotional regulation [10]. The corrected T4 sums thus clarify that T4 changed modestly, which may align with a milder involvement of the right temporal region in this specific patient sample or with individual variability in how brain fog manifests [12].

Electrode-specific conclusions

The site-specific data reinforce that C4 appears to be the most affected and, thus, most responsive electrode site in post-COVID brain fog [5]. Significant changes at F3 and P3 likewise underscore the importance of targeting multiple cortical areas during Z-score neurofeedback when addressing a multifaceted condition like brain fog. These findings resonate with prior work suggesting widespread neuroinflammation, microvasculitis, and mild hypoxia after COVID-19 may lead to dysfunction across central, temporal, frontal, and parietal regions [13,14].

Brainwave-frequency-specific effects

In addition to site-specific analyses, the present study evaluated whether the neurofeedback protocol modulated particular frequency bands (delta, theta, alpha, beta, Hi beta, beta1, beta2, beta3) relevant to brain fog symptomatology [10,15]. Table 4 summarizes the descriptive and inferential outcomes for each major frequency band.

Increases in lower frequencies: delta and theta

Delta (p = 0.046)

The decrease in the mean delta amplitude from pre- to post-treatment achieved significance, indicating that excessive slow-wave activity may have been attenuated. Although some degree of delta activity is normal (especially in sleep), heightened delta in wakeful states can correlate with reduced alertness and “cognitive dullness” [10]. A shift toward a more normative delta might help alleviate the heaviness and sluggishness often reported in brain fog.

Theta (p = 0.036)

Theta activity increased overall, which may seem counterintuitive given that too much theta can be associated with inattention. However, moderate increases in theta in specific cortical locations can reflect a transition toward a more relaxed, open, and creative cognitive state-especially when alpha also rises, and high beta decreases [11]. Indeed, the study participant described heightened focus and less mental fatigue, possibly supported by a healthier theta/alpha ratio [10].

Increases in alpha 

Alpha rises were significant (p=0.036) because alpha frequencies (8-12 Hz) reflect a relatively calm, alert, but relaxed state [10]. Individuals suffering from brain fog often exhibit disrupted alpha rhythms, which correlate with difficulty sustaining focused attention and efficient sensory integration [5]. The data show that alpha amplitude significantly increased, mirroring symptomatic improvements in attention and overall mental clarity.

Decreases in higher beta frequencies

Beta (p = 0.025)

The broad beta band (12-25 Hz) decreased significantly from pre- to post-treatment. Excessive beta activity often signifies heightened arousal, stress, anxiety, or rumination [8], which can be aggravated in post-COVID conditions [5]. Thus, lowering beta may help the brain transition from a hyper-aroused state.

Hi Beta (p = 0.012)

Reductions in Hi beta were even more pronounced, aligning with previous observations that Hi Beta (25-30 Hz) is strongly correlated with excessive mental effort, anxious rumination, and difficulties relaxing [10,15]. Clinically, decreases in Hi beta can manifest as improved calmness, diminished restlessness, and fewer intrusive thoughts.

Beta 1 (p = 0.017) and Beta 3 (p = 0.017)

These specific sub-bands of beta further confirmed the overall attenuation of high-frequency activity. Beta1 (12-15 Hz) is often considered the SMR range, which can be helpful when moderately elevated to enhance mental and motor steadiness. However, it may lose that beneficial quality if combined with excessive beta2 or beta3. Meanwhile, beta3 (18-25 Hz) is often implicated in elevated stress or cognitive overdrive [11]. The significant decreases seen here suggest successful normalization across the entire beta spectrum.

Frequency-specific conclusions

The overarching pattern-increased alpha and theta-decreased higher beta strongly indicate moving from a chronically stressed or “wired” state toward one that is calmer yet alert [10]. These shifts mirror the participant’s clinical improvements, including decreased anxiety (PASS-20, HADS-A) and partial reduction in depressive symptoms (SDS, HADS-D). Although depression did not improve to the same extent as anxiety [5], the overall qEEG changes suggest that the patient’s brain dynamics were trending in a healthier direction. This frequency rebalancing might underlie the faster-than-expected symptomatic relief often seen in Z-score protocols, even within 10-15 sessions [15].

Integrating the findings and clinical implications

Building on the original conclusion of Masuko and Masuko [5], the present data underscore that:

C4 Emerges as a Critical Site

Consistently significant changes at C4 align with the notion that brain fog can heavily impact the right central region. The combination of sensorimotor, insular, and parietal influences might drive some more salient cognitive and affective complaints [10].

Correcting T4 Totals Revises (But Does Not Negate) Temporal-Lobe Involvement

The updated T4 total (37.28 instead of 51.91) reduces the earlier overestimation of improvement at T4, shifting its percentage decrease from 24.58% to 5.02%. While T4 thus appears less dramatically changed, it remains consistent with a mild but significant temporal-lobe recalibration plausible scenario in a multifocal condition like brain fog [13].

Shifts Toward Relaxed and Efficient Neural Processing

The significant decreases in beta/Hi beta and the significant increases in alpha/theta suggest that Z-score neurofeedback helps pivot the post-COVID brain away from hyperarousal and toward optimal cortical integration [9,11]. Clinically, this likely translates into better concentration, less mental fatigue, and fewer anxiety-related symptoms despite incomplete resolution of mood-related aspects such as depression [16,17].

Fewer Required Sessions for Initial Benefit

Contrary to some neurofeedback protocols requiring 30-40 sessions to manifest changes [10,15], the participant in the Masuko and Masuko [5] case study showed immediate early improvements (e.g., alpha and theta shifts after only 1-2 sessions). This may indicate that Z-score-based multi-channel interventions can shorten the timeline for symptomatic relief, possibly due to the integrative approach of training multiple electrode sites simultaneously [8,10].

Safety and Tolerability

Congruent with other studies, no adverse effects were reported in this sample [11,15]. Transient headaches or fatigue can occur in neurofeedback, but these typically resolve quickly [5,10].

Limitations

Several limitations should be acknowledged when interpreting the findings of this secondary analysis. First, the study was based on a single-case dataset originally reported by Masuko and Masuko [5], which inherently limits the generalizability of the results. The retrospective nature of this analysis, combined with the absence of a control group, makes it difficult to definitively attribute the observed improvements solely to the Z-score neurofeedback intervention, as opposed to placebo effects or natural recovery processes. However, the repeated measure (pre-post) nature of the study adds to its validity as the subject served as its own control.

Methodologically, while the detailed electrode site-specific and frequency band analyses provided valuable insights, the reliance on a limited sample size restricted the statistical power of the Wilcoxon Signed-Rank Tests. Some electrode sites (e.g., F3 and T4) demonstrated only marginal or nonsignificant changes, raising the possibility that individual variability or measurement inconsistencies may have influenced the outcomes. Notably, the correction of the T4 pretest total, from 51.91 to 37.28, underscores potential challenges related to data accuracy and consistency, which could affect the interpretation of temporal lobe involvement in post-COVID brain fog.

Although significant shifts were observed in specific frequency bands (e.g., increased alpha and theta, decreased beta and Hi beta), the study design does not allow for the disentanglement of neurophysiological changes directly attributable to neurofeedback from those arising due to other confounding factors, such as concurrent treatments or environmental influences. The differential improvement observed across symptom domains, particularly the modest changes in depressive symptoms compared to more pronounced gains in anxiety and cognitive clarity, suggests that the intervention’s effects may be multifaceted and not uniformly distributed across all aspects of brain fog.

The short-term nature of the intervention and the absence of long-term follow-up data limit our understanding of the durability of the neurofeedback-induced changes. Without extended monitoring, it remains unclear whether these neural and clinical improvements are maintained over time or if additional sessions are required to achieve sustained benefits.

Future Research Recommendations

Building on these preliminary findings, future research should aim to address the current study’s limitations through more rigorous and expansive designs. Specifically, randomized controlled trials with larger, more diverse participant cohorts are essential to confirm the efficacy and generalizability of Z-score neurofeedback for post-COVID brain fog. Incorporating control groups-ideally with sham or alternative interventions-would help isolate the specific effects of neurofeedback from non-specific therapeutic factors.

Longitudinal studies are also recommended to assess the sustainability of neurophysiological and clinical improvements over extended periods. Such studies could provide valuable insights into optimal treatment duration, the necessity for booster sessions, and the long-term safety profile of the intervention. Additionally, future investigations should explore the mechanistic underpinnings of the observed changes by integrating complementary neuroimaging techniques, for example, functional magnetic resonance imaging (fMRI) or positron emission tomography alongside qEEG analyses. This multimodal approach may help clarify how alterations in specific electrode sites and frequency bands relate to cognitive and affective improvements.

Finally, research should consider stratifying participants based on baseline characteristics or symptom profiles to identify predictors of treatment response. A better understanding of individual variability could guide personalized neurofeedback protocols, optimizing electrode placement and frequency band targets to more effectively address the multifaceted nature of post-COVID brain fog.

## Conclusions

This secondary analysis demonstrates that Z-score qEEG neurofeedback may alleviate post-COVID-19 brain fog by normalizing brainwave activity and improving cognition and mood. Statistically significant changes were detected at electrode sites C4, F3, and P3, suggesting that these regions, linked with sensorimotor processing, executive functioning, and sensory integration, are necessary in treating brain fog. With frequency, neurofeedback served to decrease high beta, i.e., beta, Hi beta, beta 1, and beta 3, and at the same time, alpha and theta notably increased. This may suggest a change from a hyper-aroused state to a calmer, more focused brain wave pattern. The patient reported pronounced reductions in anxiety and pain, though depression showed less improvement. A revision of T4 data clarified that impacts in the right temporal region were modest rather than large but aligned with mild temporal-lobe recalibration. These findings are noteworthy, given the relatively few sessions required. Future controlled studies with standardized protocols and longer-term evaluations are essential to validate and expand upon these preliminary findings. This could establish qEEG-guided neurofeedback as a non-pharmacological treatment for persistent cognitive impairments post-COVID-19.
